# Tunable broadband plasmonic field enhancement on a graphene surface using a normal-incidence plane wave at mid-infrared frequencies

**DOI:** 10.1038/srep11195

**Published:** 2015-06-09

**Authors:** Tian Zhang, Lin Chen, Bing Wang, Xun Li

**Affiliations:** 1 Wuhan National Laboratory for Optoelectronics, Huazhong University of Science and Technology, Wuhan 430074, China; 2 Department of Electrical and Computer Engineering, McMaster University, 1280 Main Street West, Hamilton, Ontario, L8S 4L8 Canada

## Abstract

We investigate optical field enhancement for a wide mid-infrared range, originating from the excitation of graphene plasmons, by introducing a graded dielectric grating of varying period underneath a graphene monolayer. Excitation of the plasmonic mode can be achieved by illuminating a normal-incidence plane wave on the gratings due to guided-mode resonance. The gratings of varying period enable the excitation of the plasmonic mode with a very high field enhancement factor (to the order of magnitude of 1000) within a wide spectral band, which leads to the frequency-dependent spatially separated localization of the infrared spectrum modes. We also demonstrate that the excitation position of the plasmonic mode can be freely tuned by varying the thickness of the interlayer as well as the chemical potential of the graphene monolayer. This structure enables the design of two-dimensional plasmonic photonic circuits and metamaterials targeted towards numerous potential applications including optoelectronic detectors, light-harvest devices, on-chip optical interconnects, biosensors, and light-matter interactions.

The efficient concentration of optical energy into nanoscale objects and structures and the consequent enhancement of the optical field by optical antennas^1^, lightning-rod effect^2^,^3^ and nanofocusing^4^ are of great importance in various applications, including sensing^5^, tip-enhanced Raman spectroscopy^6^, and optical nonlinearity^7^. Nano-optical antennas constructed by nanoparticles or nanostructures are able to convert bulk electromagnetic radiation into strongly localized and enhanced plasmonic modes, but such antennas can only operate within a very limited bandwidth near the resonant frequencies^1^. The local optical field enhancement within a wide spectral band can be achieved using sharp metal corners or tips due to the high local charge densities^2^,^3^ and tapered plasmonic waveguide^4^,^8^. However, the above-mentioned schemes for optical field enhancement are commonly constructed using tips, tapers, wedges, grooves, and cones, which all require complicated and expensive device geometry engineering for practical application.

It would be hugely advantageous to extend the concept of energy concentration to the infrared and THz regime, which would enable significantly increased applications including probing^9^, spectroscopy^10^, and chemical analysis^11^. However, the poor optical confinement of plasmonic structures due to the negligible penetration of the electromagnetic field into the metal in low frequencies severely limits the efficiency and viability of such concepts^12^. Furthermore, owing to the significant mode mismatch, it is a great challenge to achieve the optical coupling of input radiation-either freely propagating from a laser source or delivered by an optical fiber into a nanoscale plasmonic waveguide-with high efficiency and a wide spectral band^13^.

Recent concerns regarding plasmonics have been partly shifted to graphene plasmons (GPs)^14^,^15^,^16^. Graphene, whose optical response is characterized by surface conductivity, behaves like thin metal films with negative permittivity at low frequencies via external tunability^14^,^15^, exhibiting the capacity to guide the plasmons in the infrared and THz range. In addition to wide-band tunability by varying the surface conductivity, the plasmonic waves in graphene have very large wavevectors as well as extremely high confinement, which enables us to build potential optical devices with dimensions that are significantly below the diffraction limit. Plasmonic wave localization in graphene is also followed by a strong optical field enhancement due to the significant reduction of the group velocity of the GPs^17^. Therefore, GPs can be anticipated to realize an energy concentration with a nanoscale spatial region at the infrared and THz regime, which has been proved to be a challenge for metallic nanostructures due to the highly delocalized plasmonic mode. So far, several approaches have been proposed to overcome the momentum mismatch, including graphene microribbon arrays^18^, a nano-sized metalized tip^19^, attenuated total reflection via Otto geometry^20^, and guided-mode resonances using an etched diffractive grating^21^. In this article, we investigate the optical field enhancement of plasmonic modes by introducing a graded grating with varying period underneath the graphene layer. The excitation of the plasmonic mode on the graphene surface can be achieved using a normal-incidence plane wave on diffractive gratings based on the principle of guided-mode resonance. The graded grating enables the excitation of the plasmonic mode within a wide spectral band, which leads to the frequency-dependent spatially separated localization of the infrared spectrum modes. It should be noted that some recent works on the “trapped rainbow” storage of light in metamaterials^22^,^23^ and plasmonic grating structures^10^,^16^ are closely related to the concept presented in this work.

## Results

### Direct excitation of the GPs in a graphene monolayer on a uniform diffractive grating substrate

**Figure 1:**
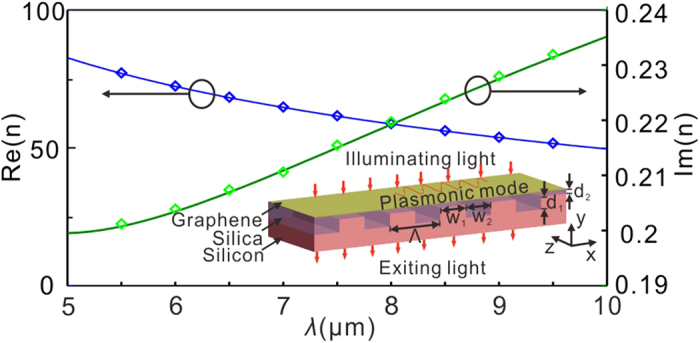
Dispersion curves of GPs in a UDGS with *μ*_c_ = 0.65 eV, *d*_2_ = 5 nm and *w*_1_ = 62 nm. The blue and green solid lines, respectively, represent the real [Re(*n*)] and imaginary [Im(*n*)] parts of the effective index (*n* = *β*/*k*_0_) of GPs, retrieved from Eq. (2), while blue and green four prisms are gotten from numerical simulations by finite-element method (FEM) based mode solver. The inset is schematics of a UDGS for excitation of GPs: a graphene monolayer on a uniform silicon/silica grating with silica as the interlayer. *Λ* is the grating period, *w*_i_ and *d*_i_ (i = 1, 2) denote the widths and thicknesses of silicon (i = 1) and silica (i = 2) layers, respectively. *w*_2_ is fixed at 62 nm in our work.

We first consider the dispersive relationship of the plasmonic mode supported by a graphene monolayer on a uniform diffractive grating substrate with a silica layer as the interlayer (UDGS, the inset of Fig. 1). In our work, the thickness of the grating layer, *d*_1_, is assumed to be 500 nm. In this case, the grating layer can be treated as infinitely thick since the silicon substrate below the grating does not affect the field distribution of the GPs in the grating/silica/graphene/air system. As the grating period is much smaller than the wavelength of the incident wave, the grating can be approximately modeled as an effective medium with the equivalent permittivity^24^
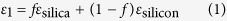
where *ε*_silica_ = 3.9 and *ε*_silicon_ = 11.9 are the respective permittivities of silica and silicon at the mid-infrared range^25^, and *f* = *w*_2_/Λ is the filling ratio of the silica.

For the four-layer grating/silica/graphene/air system, the dispersion relation of the GPs can be derived by combining the Maxwell equations and continuous boundary conditions^26^
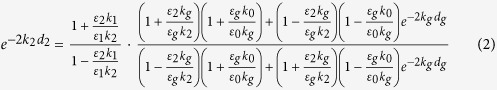
where *ε*_i_ and *k*_i_* = *(*β*^2^ − *ε*_i_*k*_0_^2^)^1/2^ (i = 1, 2, g, 0) are the permittivities and wavevectors of the grating layer (i = 1), silica (i = 2), graphene (i = g) and air (i = 0), respectively, while *β* is the propagation constant of the GPs along the x axis. The dispersion relation of the GPs by equation (2) for *μ*_c_ = 0.65 eV is represented by solid lines in Fig. 1, which agrees with the simulation results (denoted by four prisms). Owing to the large wavevector of the GPs on a graphene surface, one can theoretically predict that both of the highly confined optical field and large field enhancements due to the significant reduction of the group velocity of the GPs can be achieved simultaneously. However, only the large wavevector difference between the GPs and a free-space optical wave has to be overcome so that the unique properties of the plasmonic mode can be further exploited. As for the excitation of the GPs on the graphene monolayer shown in the inset of Fig. 1, the grating period Λ should satisfy the phase matching equation
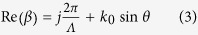
where *j* is the diffraction order and *θ* is the incident angle. To excite the plasmonic waves with wavelength *λ* and the fundamental order *j* = 1 under the condition of normal incidence *θ* = 0, the following expression should be satisfied
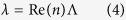


**Figure 2:**
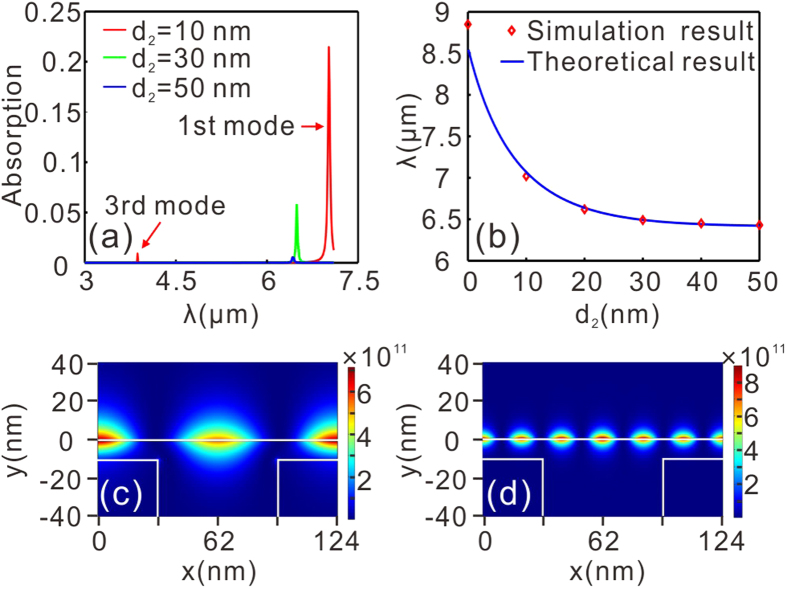
(**a**) Absorption spectrums with *d*_2_ = 10 nm (red line), 30 nm (green line) and 50 nm (blue line) from numerical simulations by COMSOL. The two absorption peaks for *d*_2_ = 10 nm correspond to fundamental (*λ* = 7.02 μm) and third-order (*λ* = 3.87 μm) plasmonic modes, respectively. (**b**) The excitation wavelengths of fundamental plasmonic mode versus *d*_2_, where the solid line and dotted lines are obtained from Eq. (4) and FEM simulation, respectively. |E_x_|^2^ distribution of the fundamental (**c**) and third-order (**d**) plasmonic modes, corresponding to the two peaks in (**a**). The value of |E_x_|^2^ of incident wave is 6.07 × 10^9^ V^2^/m^2^. The white solid lines show the outlines of grating and graphene. For the UDGS, *μ*_c_ = 0.65 eV and *w*_1_ = 62 nm.

**Figure 3:**
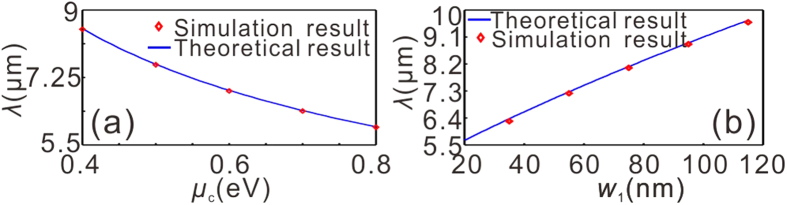
(**a**) Dependence of the excitation wavelength *λ* of the fundamental plasmonic mode on *μ*_c_ with *d*_2_ = 20 nm and *w*_1_ = 62 nm. (**b**) Dependence of the excitation wavelength, *λ*, of the fundamental plasmonic mode on *w*_1_ with *d*_2_ = 5 nm and *μ*_c_ = 0.65 eV. The solid lines and red four prisms are extracted from Eq. (4) and numerical simulations, respectively.

Figure 2 shows the optical response of the UDGS for different thicknesses of the silica layer, *d*_2_, with *μ*_c_ = 0.65 eV and *w*_1_ = 62 nm. It can be clearly seen from Fig. 2(a) that there exist two absorption peaks for *d*_2_ = 10 nm, which can be attributed to the excitation of the fundamental (*j* = 1) and third-order (*j* = 3) plasmonic modes on the graphene surface. The missing second-order (*j* = 2) mode is here caused by the vanishing dipole moment. The scaling of the resonant wavelength, associated with the grating period, is consistent with equation (3). The absorption peaks become weaker and weaker as *d*_2_ increases due to the fact that the overlap between the GPs and the grating reduces as *d*_2_ increases and hence reduces the excitation efficiency. The scaling of the fundamental-mode resonant wavelength with respect to the thickness of the silica layer, *d*_2_, by finite element method (FEM) simulation agrees with the theoretical results of equation (4), as shown in Fig. 2(b). It is also interesting to note that the value of the resonant wavelength decreases with the increased *d*_2_. This is because the effective index of the plasmonic mode in the grating/silica/graphene/air system reduces with the increased thickness of the silica layer, *d*_2_. From Figs 2(c,d), we can also see a greatly enhanced plasmonic field on the graphene surface due to the significant reduction of the group velocity of the GPs, while the square of the electric field is approximately 1146 [Fig. 2(c), fundamental mode] and 137 [Fig. 2(d), third-order mode] times larger than the illuminating plane wave. We define the electric field enhancement factor as the ratio of the electric field intensity on the graphene surface (|E|^2^) and light source (|E_0_|^2^). The enhancement level presented here is quantitatively comparable to that reported in plasmonic nanocavity gratings made of nanogrooves in a gold film^27^. The resonant wavelength decreases with the increased chemical potential for a given grating period [Fig. 3(a)], and increases with the grating period for a fixed chemical potential [Fig. 3(b)]. In both cases, the theoretical prediction significantly agrees with the simulation result.

### Direct excitation of broadband GPs in a monolayer graphene on a graded diffractive grating substrate

**Figure 4:**
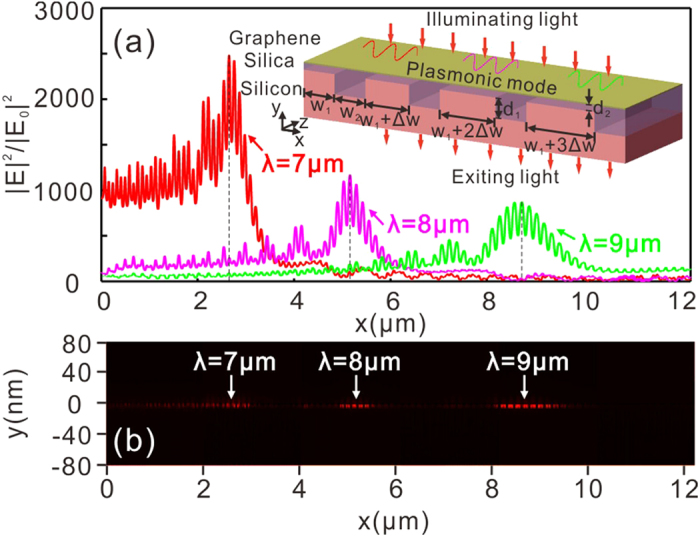
(**a**) Electric field enhancement (E|^2^/|E_0_|^2^|) on the graphene surface of the structure with *μ*_c_ = 0.65 eV, *d*_2_ = 5 nm for three different incident wavelengths. (**b**) Corresponding distributions of the square of the electric field (|E|^2^) for three wavelengths in the x-y plane. The inset in (**a**) is a schematic of a GDGS for excitation of GPs within a wide spectral band: a graphene monolayer on a graded diffractive grating substrate with silica as the interlayer. The width of silicon layer increases linearly from 20 nm to 115 nm with a step of Δ*w* = 1 nm and the width of the whole structure along x axis is 12.3 um. In the simulation, perfect electric conductor boundary condition is chosen.

The UDGS can only excite the GPs on a graphene monolayer within a very narrow bandwidth around the resonant frequency. Bearing in mind that the resonant frequency is dependent on the grating period [Fig. 3(b)], we further propose a graphene monolayer on a graded diffractive grating substrate (GDGS, the inset of Fig. 4) to enlarge the spectral bandwidth of the plasmonic mode. The graded grating is achieved by linearly increasing the width of the silicon layer, *w*_1_, along the x direction, while keeping the width of the silica layer, *w*_2_, constant. Wide-band plasmonic field enhancement can be anticipated under the illumination of a normal-incidence plane wave. The simulation result shown in Fig. 4 demonstrates that the GPs of different wavelengths are enhanced at different positions on the graphene surface, leading to a plasmonic “trapped rainbow” effect. It should be noted that the present “trapped rainbow” effect is based on the correlative relationship between the excitation wavelength of the plasmonic mode and the grating period due to the guided-wave resonance. This working principle is different from the previously proposed graded-grating based plasmonic waveguides for the “trapped rainbow” effect^10^,^16^, where the frequency-dependent spatially separated localization of the plasmonic wave is achieved by adiabatically reducing the group velocity of the plasmonic wave along the propagation direction. Meanwhile, efficient excitation of the GPs can be easily achieved using a normal-incidence plane wave, while it remains very challenging for that in Ref.^16^. It can also be seen from Fig. 4(a) that the electric field enhancement factor is quantitatively comparable to that for a UDGS and reaches a maximum of 7 um, more than 2000 times larger than that of the incident plane wave. It should be emphasized that the electric field enhancement factor (|E|^2^/|E_0_|^2^) is independent of the power enhancement on the graphene surface, which is proportional to the power of incident light. The average electric field enhancement for the first 3 um in the left region is approximately 1000, but this does not mean the power enhancement is larger than 1 as the power of the incident light source is unchanged. The GPs excited by the silicon grating propagate along the graphene surface and hence suffer an absorption loss from the graphene monolayer. This is why the electric field intensity reaches its maximum value at the center position for each excitation wavelength [vertical dotted lines in Fig. 4(a)], associated with the excitation period of the grating, but shows a gradual reduction as it departs from that center position. However, since the excited GPs will propagate toward both sides, while the right-going plasmon, whose frequency is approaching the cutoff frequency, suffers a larger propagation loss than the left-going one, it is not surprising that the electric field enhancement factor decays more slowly (with higher electric field enhancement) in the left region than in the right region. It should be noted that the field enhancement for *λ* = 7 μm is much stronger than that for the other two wavelengths (i.e. 8 μm and 9 μm), even in the first 0 ~ 2 μm region. The reason for this can be explained as follows. The plasmon field enhancement at the excitation position for *λ* = 7 μm is strongest among the three wavelengths as it is validated by the simulation results. The left-going one will experience a GDGS, whose grating period reduces along the propagation direction. A GDGS can be deemed to be a series of uniform UDGS with a constant period, whose propagation loss can be retrieved from the imaginary part of the propagation constant [Im(*β*) = 2πIm(*n*)/*λ*]. The calculated results show that the propagation loss by a UDGS is almost equal for the three wavelengths. For example, the values of Im(*β*) with *λ* = 7, 8 and 9 μm at *w*_1_ = 20 nm are 1.81 × 10^5^, 1.62 × 10^5^ and 1.46 × 10^5^ m^−1^, respectively. Additionally, the left-going plasmon for a longer wavelength undergoes a much larger propagation distance between the excitation position and the 0 ~ 2 μm region. As a result, the field enhancement for *λ* = 7 μm is higher than that for a longer wavelength in the first 0 ~ 2 μm region.

**Figure 5:**
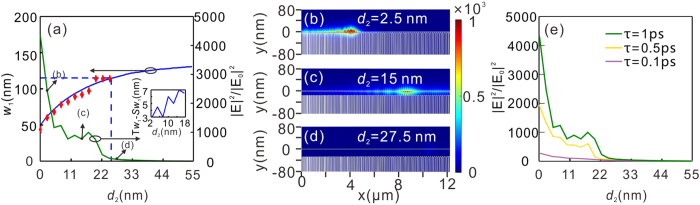
(**a**) Dependence of the excitation positions, represented by *w*_1_, and electric field enhancement |E|^2^/|E_0_|^2^ on *d*_2_. The solid blue lines and red marks “Φ” are retrieved from Eq. (4) and numerical simulations, respectively. The horizontal and vertical blue dashed lines, respectively, represent the value of *w*_1_, and *d*_2_ for the rightmost grating of the GDGS. The inset shows the difference of *w*_1_ between the theoretical value and used value in our design versus *d*_2_. (**b**–**d**) The distribution of normalized electric field intensity (|E|^2^/|E_0_|^2^) with different gap separations: *d*_2_ = 2.5 nm (**b**), 15 nm (**c**), and 27.5 nm (**d**). (**e**) The electric field enhancement |E|^2^/|E_0_|^2^ varies with *d*_2_ with three values of *τ*. In the simulations, a TM plane wave of *λ* = 8 μm is illuminating the GPGS with Δ*w* = 1 nm, while the chemical potential of *μ*_c_ is equal to 0.65 eV.

We then consider the influence of the interlayer between the graphene monolayer and the grating on the optical properties of the GPs on the graphene surface, which can be achieved by changing the gap separation, *d*_2_. According to equation (4), for a fixed illumination wavelength (*λ* = 8 μm in our case), a much wider *w*_1_ is required to excite the GPs as *d*_2_ increases due to the gradual reduction of the value of *n* [Fig. 5(a)]. The electric field distribution shown in Figs 5(b,d) numerically demonstrates that the position of the enhanced GPs can be varied for a given wavelength associated with the gap separations. However, the overlap between the GPs and the grating is reduced as *d*_2_ increases, which hence reduces the effective strength of the grating. Therefore, it is not surprising that the electric field enhancement tends to decrease as *d*_2_ increases. The electric field enhancement can reach 4336 when *d*_2_ = 0 nm. The existence of a certain fluctuation in the enhancement for *d*_2,_ in the range of 2.5 nm to 17.5 nm [green solid line in Fig. 5(a)] can be attributed to the fluctuating excitation efficiency of the GPs. As *d*_2_ increases from 2.5 nm to 17.5 nm with the step of 2.5 nm, the value of *w*_1_ that is used in our design is never exactly equal to the theoretical value [see the inset of Fig. 5(a)]. Moreover, the difference between the two values shows a fluctuation, which finally results in a fluctuation in the field enhancement. It is also interesting to note that the field enhancement is sharply reduced as *d*_2_ is further enhanced beyond 26 nm [denoted by the vertical dashed line in Fig. 5(a)]. This is because there does not exist any grating in the GDGS that is capable of exciting the GPs, even for the rightmost grating [see the electric field distributions for *d*_2_ = 27.5 nm in Fig. 5(d)]. In addition, we have also estimated the electric field enhancements with the scattering time of 0.1 and 0.5 ps, which more conservatively reflect the practical transport loss of graphene [Fig. 5(e)]. The resultant electric field enhancement still remains high, especially for τ = 0.5 ps. The maximum value of the electric field enhancement at *d*_2_ = 0 with τ = 0.5 ps is 1942, approximately 2.2 times smaller than that with τ = 1 ps (4336). The electric field enhancement will decrease to several hundred when the scattering time is further reduced to 0.1 ps. This enhancement level is still quantitatively comparable to that found with previously reported plasmonic nanocavity gratings^27^.

It should be noted that, for future tuning of the position of the GPs, one feasible approach might be the use of a piezoelectric material as the dielectric interlayer, whose thickness could be temporally modulated by an external electric field. Alternatively, we could also employ a liquid crystal as the interlayer, whose optical properties could be modulated via an external voltage.

**Figure 6:**
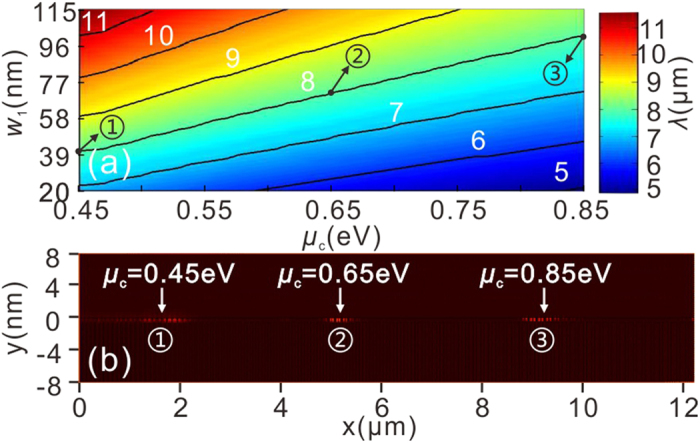
(**a**) Influence of *w*_1_ and *μ*_c_ on the excitation wavelength, *λ*, of GPs for the GDGS. The solid curves labeled with numbers depict the contour lines of *λ* in the *w*_1_-*μ*_c_ plane. (**b**) Corresponding distributions of the square of the electric field (|E|^2^) for three values of the chemical potentials as the excitation wavelength is fixed at 8 μm, while all the other geometrical parameters are the same as those of Fig. 5(b).

In addition, the position of the GPs could be dynamically tuned by varying the graphene’s optical property by external field. This unique feature renders graphene promising for constructing novel active nanophotonic devices^14^,^16^,^18^,^28^. Figure 6(a) shows the influence of *w*_1_ and *μ*_*c*_ on the excitation wavelength of the GPs for the GDGS in the inset of Fig. 4. Considering that a doping level of 6 × 10^13^ cm^−2^, corresponding to a Fermi-level of *μ*_*c*_ = 0.9 eV, has been achieved experimentally^29^,^30^, the assumed range of the chemical potential (0.45–0.85 eV) is realistic. In Fig. 6(a), the value of the excitation wavelength of the GPs, *λ*, undergoes a blue shift as *μ*_*c*_ increases for a given *w*_1_. The solid curves labeled with numbers depict the contour lines of *λ* in the *w*_1_-*μ*_*c*_ plane. What makes the present “trapping rainbow” structure unique, as opposed to previous proposals^10^,^16^, is that the “trapping” position for each frequency can be shifted simply by varying the chemical potential [Fig. 6(b)].

## Discussion

In conclusion, we have presented a proposal for optical field enhancement for a wide mid-infrared range by exciting the plasmonic mode with a graphene monolayer on a graded grating with varying period. Excitation of the plasmonic mode can be realized simply by illuminating a TM polarized plane wave with a normal-incidence on the graded gratings. Since the excitation wavelength is dependent on the grating period, plasmonic waves with different wavelengths can be enhanced at different positions on the graphene surface, associated with the grating period, leading to the plasmonic “rainbow trapping” effect^10^,^16^,^22^,^23^. The working principle is quite different from those structures used for the “trapped rainbow” effect based on metamaterials and plasmonics^10^,^16^,^22^,^23^, where the frequency-dependent spatially separated localization of the plasmonic wave is achieved by adiabatically reducing the group velocity of the electromagnetic wave along the propagation direction. We also demonstrate that the excitation position of the plasmonic mode can be freely tuned by varying the thickness of the interlayer as well as the chemical potential of the graphene monolayer. Numerical simulation results concerning the optical properties of the structure are consistent with the theoretical prediction. The present results indicate a significant number of potential applications in graphene-based optical devices, such as optoelectronic detectors, light-harvest devices, on-chip optical interconnects, biosensors, and light-matter interactions.

## Methods

The optical properties of graphene are typically described by its surface conductivity. The Kubo formula has been widely employed to retrieve its surface conductivity in a series of theoretical and experimental studies on graphene^31^,^32^,^33^
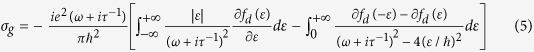
where *f*_d_ = 1/(1 + exp[(*ε* − *μ*_c_)/(*k*_B_*T*)]) is the Fermi-Dirac distribution, *ε* is the energy, *μ*_c_ is the chemical potential, *T* is the temperature, *e* is the electron charge, 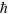 is the reduced Planck’s constant, *k*_B_ is the Boltzmann constant, *ω* is the radian frequency, and *τ* is the momentum relaxation time representing the loss mechanism due to the carrier intraband scattering.

In the mid-infrared range with |*μ*_c_| ≫ *k*_B_*T*, the surface conductivity of graphene could be approximated as^32^
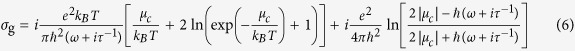


In our study, *T* = 300 K and *τ* = 1 ps. Considering that in high-quality suspended graphene a DC mobility of μ > 100000 cm^2^V^−1^s^−1^ has been experimentally achieved^34^, which leads to *τ* > 1.5 ps, our setting of *τ* = 1 ps can be said to conservatively reflect the practical transport loss of graphene. However, as a graphene monolayer with a carrier mobility of more than 100000 cm^2^V^−1^s^−1^ is rarely seen in graphene samples in practice, we have also studied the electric field enhancement utilizing a much more realistic scattering time of graphene [see Fig. 5(c)].

By treating the graphene monolayer as an ultra-thin metal film, graphene’s equivalent permittivity can be written as^14^
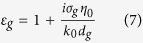
where *η*_0_ (≈377 Ω) is the impedance of air, *k*_0_ is the light wavevector in free space, and *d*_g_ is the thickness of the graphene monolayer (assumed to be 0.5 nm in our work).

To calculate the dispersion relation of the GPs, two-dimensional FEM simulation based on the mode solver of COMSOL is employed. In the simulation, 5-layer meshes are employed to denote the graphene layer while non-uniform meshes with a maximum element size of 500 nm are adopted to represent the other regions besides graphene.

## Additional Information

**How to cite this article**: Zhang, T. *et al.* Tunable broadband plasmonic field enhancement on a graphene surface using a normal-incidence plane wave at mid-infrared frequencies. *Sci. Rep.* **5**, 11195; doi: 10.1038/srep11195 (2015).
